# A Beta Mixture Model for Careless Respondent Detection in Visual Analogue Scale Data

**DOI:** 10.1017/psy.2025.10041

**Published:** 2025-09-23

**Authors:** Lijin Zhang, Benjamin W. Domingue, Leonie V. D. E. Vogelsmeier, Esther Ulitzsch

**Affiliations:** 1 Graduate School of Education, Stanford University, Stanford, CA, USA; 2 Department of Methodology, Tilburg University, Tilburg, The Netherlands; 3 Centre for Educational Measurement (CEMO), University of Oslo, Oslo, Norway; 4 Centre for Research on Equality in Education (CREATE), University of Oslo, Oslo, Norway

**Keywords:** careless respondents, mixture modeling, visual analogue scale (VAS)

## Abstract

Visual Analogue scales (VASs) are increasingly popular in psychological, social, and medical research. However, VASs can also be more demanding for respondents, potentially leading to quicker disengagement and a higher risk of careless responding. Existing mixture modeling approaches for careless response detection have so far only been available for Likert-type and unbounded continuous data but have not been tailored to VAS data. This study introduces and evaluates a model-based approach specifically designed to detect and account for careless respondents in VAS data. We integrate existing measurement models for VASs with mixture item response theory models for identifying and modeling careless responding. Simulation results show that the proposed model effectively detects careless responding and recovers key parameters. We illustrate the model’s potential for identifying and accounting for careless responding using real data from both VASs and Likert scales. First, we show how the model can be used to compare careless responding across different scale types, revealing a higher proportion of careless respondents in VAS compared to Likert scale data. Second, we demonstrate that item parameters from the proposed model exhibit improved psychometric properties compared to those from a model that ignores careless responding. These findings underscore the model’s potential to enhance data quality by identifying and addressing careless responding.

## 1 Introduction

**Figure 1:**
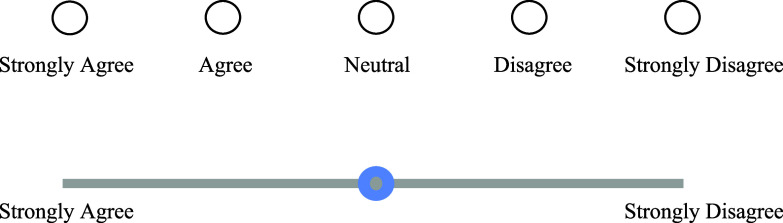
Likert scale and VAS.

Self-report scales are widely used in social science to measure latent constructs. To effectively capture the underlying constructs, selecting an appropriate scale format is crucial. Throughout the last decades, Likert-type scales have been established as the “go-to” standard for research in psychology, social, and educational sciences (Jebb et al., 2021). Likert scales involve selecting discrete ordinal categories (e.g., from strong agreement to strong disagreement), which can simplify the response process (Likert, 1932). However, this format may also restrict the range of responses, limiting the ability to capture emotions, attitudes, and beliefs in a more nuanced way. Visual Analogue scales (VASs) are an increasingly popular alternative to Likert-type scales. A VAS (Hayes & Patterson, 1921) allows respondents to indicate their feelings on a continuous line (Figure 1), which may capture nuance that Likert scales might overlook, providing a more detailed measurement of attitudes or emotions. For instance, research has demonstrated that VASs are more effective in detecting subtle changes in pain perception and are less prone to confounding factors and ceiling effects than Likert scales, particularly in the context of measuring patient satisfaction (Price et al., 1994; Voutilainen et al., 2016). It is, therefore, not surprising that the utilization of VASs in applied psychological, social science, and clinical research has expanded extensively in recent years (Åström et al., 2023; Haslbeck et al., 2024; Kuhlmann et al., 2017; Sung & Wu, 2018).

Psychometric research on VASs, however, has not kept pace with this increased usage in applied settings, and standard psychometric approaches to evaluate important aspects of VAS data quality are lacking. The present study aims to begin filling this gap. We do so by focusing on the detection of responding due to careless or insufficient effort—i.e., providing responses without investing effort into carefully evaluating the administered items and leaving data contaminated with responses that do not reflect what researchers want to measure (Huang et al., 2015)—as a crucial aspect of ensuring data quality. To provide an approach that is tailored to detecting careless responding in VAS data, we integrate existing measurement models for VASs (Noel & Dauvier, 2007) with mixture item response theory (IRT) or factor models for identifying and modeling careless responding. Such developments have previously been limited to Likert-type scales (Uglanova et al., 2025; Ulitzsch, Pohl, et al., 2022; Ulitzsch, Yildirim-Erbasli, et al., 2022; Ulitzsch, Pohl, et al., 2024; van Laar & Braeken, 2022) and unbounded continuous response data (e.g., normally distributed with infinite range) (Arias et al., 2020; Kam & Cheung, 2023; Zhang et al., 2025). Our proposed mixture modeling approach not only facilitates gauging VAS data quality and accounting for careless responding when drawing conclusions on constructs measured with VASs but also opens the path to comparative analyses of the occurrence of careless responding across different scale formats.

The remainder of the article is structured as follows: First, we briefly discuss VASs and their usage in psychological, social, and clinical science research. Second, we review existing approaches to the detection of careless responding, which, so far, have predominantly been targeted to Likert-type scales or continuous, unbounded data. To expand these previous approaches to identifying careless responding in VAS settings, we combine them with existing measurement models for VASs, which are reviewed next. After presenting the proposed model, we evaluate its parameter recovery under realistic research settings in a simulation study. We also demonstrate through a simulation study why mixture modeling designed for unbounded continuous data can be unsuitable for VAS data. In an empirical application to data administering the same scales with VAS and Likert-type scale formats, we further illustrate the real-world application of the proposed model and provide initial insights into differences in careless responding across these different scale types.

### 1.1 VASs

VASs provide a straightforward method for respondents to indicate their position along a continuum. Typically, a VAS includes a line with two anchors at either end. These anchors usually feature verbal descriptors representing opposite ends of a semantic dimension (e.g., “strongly agree” and “strongly disagree,” Figure 1). However, anchors can also be visual or auditory, including pictures or sound clips. For example, visual cues, such as smiley faces, can be used to help participants, such as young children, who may not fully understand verbal descriptions, indicate their feelings (Reips & Funke, 2008).

Several advantages of VASs are discussed in the literature. One key benefit is their high sensitivity to variations in the latent construct, allowing for the detection of subtle shifts in perceptions or emotional states, which may be missed by Likert-type scales (Price et al., 1994). Another advantage is that, unlike Likert-type scales—whose psychometric properties can be heavily influenced by the number of response categories and for which selecting an appropriate number is not straightforward—VASs entirely avoid such issues (see, e.g., Kutscher & Eid, 2024, for a discussion). The unique characteristics of VASs make it a valuable tool in both academic and clinical settings, where precise measurement of continuous variables is essential. In clinical research, for instance, VAS has proven effective for assessing patient pain levels (McCormack et al., 1988; Myles et al., 2017; Price et al., 1994). Similarly, in psychological research, VASs have been widely applied to measure constructs of interest, such as emotional states (Askim & Knardahl, 2021; Zhou & Chen, 2009), self-esteem (Brumfitt & Sheeran, 1999), and quality of life (Weigl & Forstner, 2021).

Nevertheless, VASs may also be more demanding to use and could lead to quicker disengagement (Haslbeck et al., 2024), potentially affecting data quality as a result. Comparing Likert-type scales and VASs in a children’s sample, Van Laerhoven et al. (2004), for instance, found higher rates of missing responses in VAS data as well as self-reported preferences and higher ease of use for the Likert-type scale. Thorough comparisons of whether or not VASs and Likert-type scales elicit respondent disengagement to a different extent and, as such, provide data of different quality, however, are lacking. In this study, we provide a tool that facilitates filling this gap.

### 1.2 Careless responding and its detection in Likert-type scales

In collecting and investigating survey data, the detection of careless respondents is crucial to ensure the trustworthiness of conclusions drawn from self-report data (Meade & Craig, 2012). Careless and insufficient effort responding occurs when participants fail to engage with the content of the survey questions properly, e.g., by not reading them carefully, rushing through responses, or being distracted (Meade & Craig, 2012). This phenomenon is widespread in research relying on self-report data, with studies indicating that careless response rates can range from 1% up to 50% (Douglas et al., 2023; Ward & Meade, 2023). Such careless responses are problematic because they do not accurately reflect the latent variables intended to be measured. Consequently, careless and insufficient effort can heavily distort results, potentially diminishing estimates of reliability and construct validity, comprising factor analysis outcomes, and leading to erroneous conclusions on relationships among constructs of interest (Ward & Meade, 2023).

Previous developments in careless respondent detection have predominantly focused on Likert scale data, most commonly drawing on response-pattern-based indicators like the long string index and Mahalanobis distance. The long string index flags excessive uniformity by measuring the number of consecutive identical responses, while Mahalanobis distance detects unusual response patterns conceptualized as multivariate outliers. The application of such indicators to VAS data, however, is not always straightforward. For instance, while Mahalanobis distance could be adaptable for use with VAS data, using the long string index poses challenges. Since VAS responses are continuous rather than discrete, defining “identical” responses is difficult, and small fluctuations in consecutive values can obscure the detection of inattentive patterns.

Alongside these indicator-based approaches, confirmatory mixture IRT or factor models have recently been proposed to detect inattentive or hasty responses by analyzing item responses and, if available, collateral information on respondent behavior as contained in response times (Arias et al., 2020; Kam & Cheung, 2023; Ulitzsch, Pohl, et al., 2022; Ulitzsch, Yildirim-Erbasli, et al., 2022; van Laar & Braeken, 2022; Zhang et al., 2025). The overarching principle of these confirmatory mixture modeling approaches to careless respondent detection is the translation of theoretical considerations on respondent behavior into two mixture component models—one representing an assumed attentive and the other one an assumed careless data-generating process. Attentive item responses are assumed to reflect the to-be-measured traits, i.e., to follow standard IRT or confirmatory factor analysis (CFA) measurement models (Arias et al., 2020; Roman et al., 2024; Ulitzsch, Pohl, et al., 2022; Ulitzsch, Yildirim-Erbasli, et al., 2022; Ulitzsch, Pohl, et al., 2024). Careless item responses, in contrast, are assumed to be driven by respondents’ category preferences (Arias et al., 2020) or to be random (van Laar & Braeken, 2022). These models have proven effective in accurately identifying careless respondents and mitigating biases in survey results, thereby enhancing the reliability of the collected data and ensuring the validity of conclusions drawn from them. However, factor mixture models suggested for identifying careless respondents in continuous data (Kam & Cheung, 2023; Roman et al., 2024; Zhang et al., 2025) are not directly applicable to VAS data due to their unique continuous and bounded nature (Noel & Dauvier, 2007). A key challenge is that bounded responses often display a skewed distribution (Noel & Dauvier, 2007; Verkuilen & Smithson, 2012), rendering the normal distribution assumptions in CFA measurement models inappropriate for modeling. This issue may be further exacerbated by careless responding, which may, as illustrated below, result in distributions that heavily deviate from normality. The aim of the present study is, therefore, to provide a confirmatory mixture modeling approach to careless respondent detection that is tailored to VAS data. To this end, we expand the Beta item response model (IRM) proposed by Noel & Dauvier (2007) by a mixture component for flexibly absorbing careless response patterns on VASs.

### 1.3 The Beta IRM (Noel & Dauvier, 2007)

Data from the VASs is typically converted into numerical values ranging from 0 to 1, representing the percentage of the distance from one endpoint to the selected position. While this data is continuous, its bounded nature poses challenges for traditional models such as CFA. To address this, Noel & Dauvier (2007) introduced the Beta IRM. This model draws on the Beta distribution (Ferrari & Cribari-Neto, 2004) to analyze continuous bounded responses. The Beta distribution is defined on the interval (0, 1), making it suitable for modeling data that is similarly bounded.

The Beta IRM defines the response variable 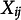 of respondent 
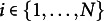
 provided to item 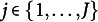 as a Beta-distributed random variable, 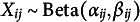 , where the parameters 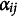 and 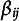 might be interpreted as “acceptance” and “refusal” parameters. The “acceptance” parameter 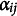 signifies the inclination toward the higher end of the scale (closer to 1), while the 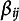 parameter signifies the inclination toward the lower end of the scale (closer to 0). In the multidimensional extension for measuring dimension 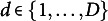 , assuming a simple structure, the parameters 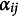 and 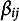 are modeled as functions of the latent trait 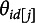 , which represents respondent *i* ’s location on the *d*th ( 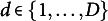 ) dimension measured by item *j*, as well as the item wording 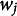 , item difficulty 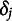 , and item dispersion 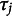 : 
(1)

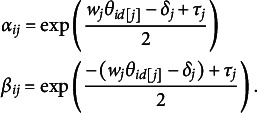

Note that the function 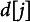 maps item *j* to the latent dimension *d* it is designed to assess. The item wording 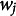 is pre-specified, where 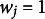 indicates that item *j* is positively worded, and 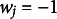 denotes a negatively worded item. The remaining item parameters, 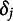 and 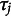 , are freely estimated. The item dispersion parameter 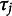 governs the response variability, with higher values indicating lower variance, conditional on the latent trait and item difficulty. For model identification, the means of the latent traits are set to zero.

The expected value of the response variable 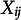 and the probability density function of its realization 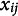 are then: 
(2)

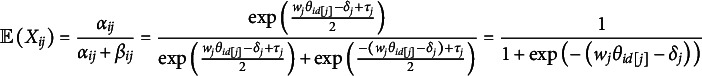



(3)

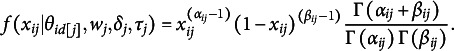

Note that 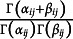 is the normalizing constant of the Beta distribution, ensuring the probability density integrates to 1 over the interval (0, 1).

**Figure 2:**
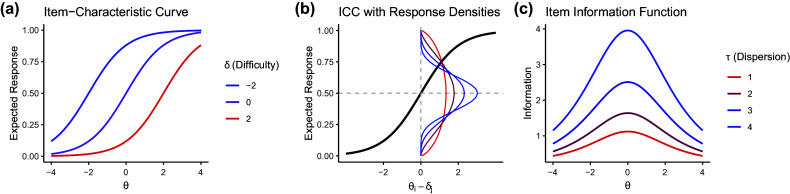
Beta IRM. *Note*: (a) ICC for different difficulty parameters, with dispersion fixed at 0. (b) ICC with response densities for 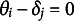 under different dispersion parameters. (c) Item Information Function for different dispersion parameters, with difficulty fixed at 0.

The logistic form of the expected response function shows the familiar S-shaped curve, which is commonly used in IRT to model the probability of item endorsement as a function of person and item parameters. To illustrate the item parameters, we focus on the condition with a unidimensional latent factor ( 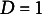 ) and positively worded items ( 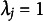 ) to demonstrate how variations in item difficulty 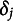 and dispersion 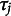 influence the expected response and item information. Figure 2a demonstrates the role of the item difficulty parameter, which determines the location of the item characteristic curve (ICC) along the scale of the latent trait. The 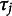 parameter represents the dispersion or precision of the item. It does not influence the expected responses (Eq 2); however, it enables different items to exhibit different response variabilities around the expected value. A higher 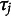 value results in a more peaked response density for a given level of 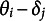 , as well as a more peaked information function which indicates that the item provides more information (precision) at certain trait levels (Figure 2b and c. Details regarding the Fisher information function of the Beta IRM can be found in Noel & Dauvier (2007)).

The Beta IRM provides several advantages. First, it accurately captures the bounded nature of VAS data. VAS data are sometimes analyzed with traditional factor analysis with normality assumptions (Wortmann et al., 2021). These, however, may not hold for bounded data, especially when responses are skewed or clustered near the boundaries. The Beta IRM avoids this limitation, providing a more flexible and accurate framework for analyzing such data. Second, the model offers a straightforward interpretation of the parameters, as they directly relate to the person’s trait location and item difficulty, similar to traditional IRT models. Third, the logistic form of the expected response function allows for easy comparison with existing IRT models, facilitating a seamless integration of the model into existing psychometric frameworks. Overall, the Beta IRM represents a significant advancement in the analysis of VAS data, offering a theoretically sound and practically viable solution for handling the unique challenges posed by continuous bounded responses (Noel & Dauvier, 2007).

## 2 The proposed model

**Figure 3:**
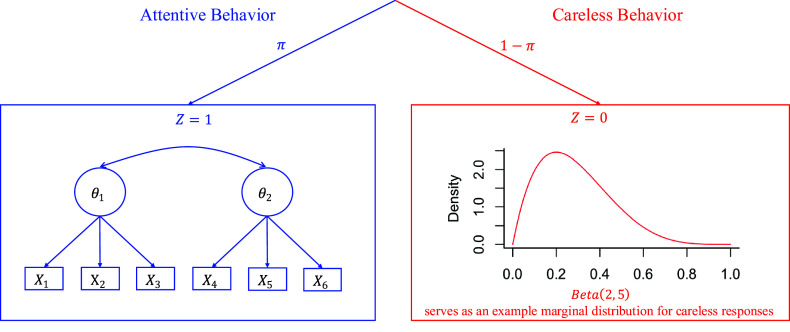
Model structure. *Note*: For attentive respondents, responses are assumed to follow a Beta IRM, governed by item and person parameters; a two-factor, six-item structure is used as an example. For careless respondents, responses are assumed to stem from a common Beta distribution, with Beta(2, 5) used as an illustrative example in the figure.

Building upon the Beta IRM (Noel & Dauvier, 2007), we propose an extended Beta Mixture IRM to identify careless respondents in VAS data. Adapting concepts from previous confirmatory mixture models (Arias et al., 2020; Kam & Cheung, 2023; Ulitzsch, Pohl, et al., 2022; Ulitzsch, Yildirim-Erbasli, et al., 2022; van Laar & Braeken, 2022), we classify respondents into attentive ( 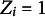 ) and careless ( 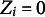 ) groups based on their response patterns’ alignment with measurement models formulated to capture different types of respondent behavior. The proposed model is expressed as follows (Figure 3): 
(4)

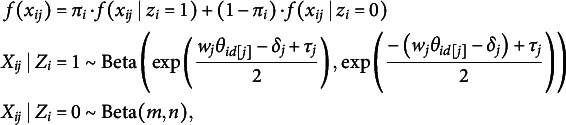

where 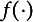 denotes a general probability density function, and 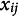 represents the realization of the continuous bounded response from respondent *i* to item *j*. The latent variable 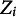 indicates class membership, where 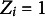 denotes the attentive class, and 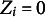 denotes the careless class, and 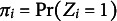 represents the probability that individual *i* belongs to the attentive class. The response distribution is a mixture of two components. The first component, 
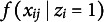
 , models responses for attentive individuals as a Beta distribution governed by the person’s latent trait levels 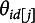 , item wording 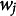 , item difficulty level 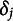 , and the dispersion parameter 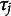 . To facilitate disentangling careless from attentive responding, we recommend modeling raw responses obtained from both positively and negatively worded items (as in Kam & Cheung, 2023; Ulitzsch, Yildirim-Erbasli, et al., 2022; Vogelsmeier et al., 2024). Information on item wording is incorporated into the model through the parameter 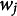 , which takes the value 1 or -1.

The second component, 
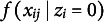
 , models responses for careless individuals using a Beta distribution with common parameters 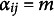 and 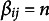 that generalize across all items and persons. This reflects the assumption that careless respondents do not process item content, and that, hence, the selected responses should neither be governed by respondent’s trait levels nor impacted by item characteristics.

**Figure 4:**
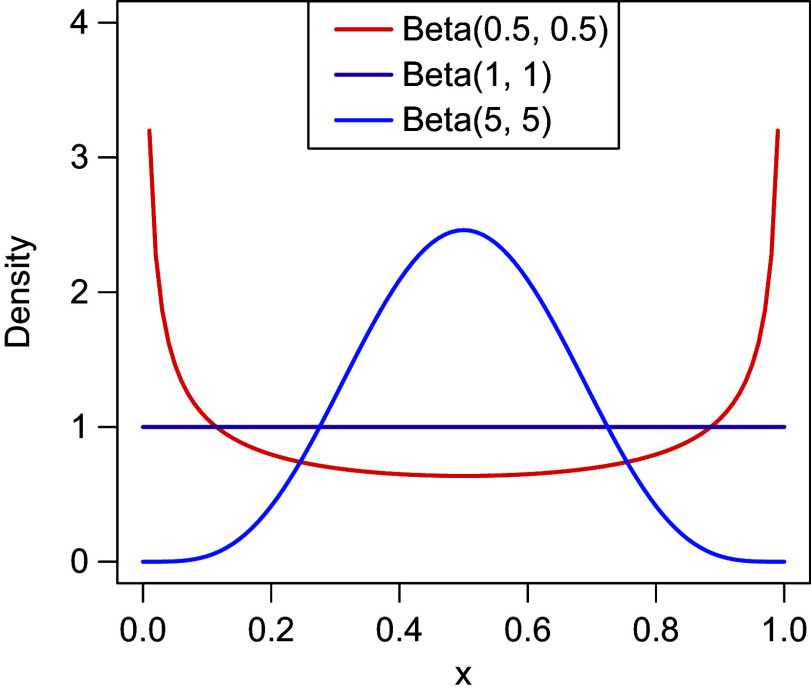
Density plot for different Beta distributions.

The Beta distribution of the careless component is assumed to “absorb” different careless response patterns; hence, its parameters will reflect the marginal distribution over all types of careless response patterns in the population (see Ulitzsch, Pohl, et al., 2022), approximated by a Beta distribution. The unstructured Beta distribution assumed for the careless class offers a flexible means of capturing the marginal distribution of diverse careless behaviors in the population. For example, a 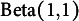 distribution suggests marginally uniform selection across the scale, 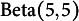 indicates a marginal tendency to select around the midpoint, and 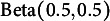 reflects marginal preferences for the opposite ends of the slider (Figure 4).

We acknowledge that careless behaviors may take various forms and may differ across respondents (e.g., some may position their slider randomly, while others may move their slider toward one end of the scale). As such, the careless component model will most likely be misspecified. However, since the particular type(s) of careless responding displayed are typically not of substantive interest, we do not aim to disentangle possibly different subtypes of careless behavior or to interpret the internal structure of the careless class. Instead, the careless component of the mixture model is conceptualized as a residual class with minimal structure, designed to capture any response pattern that systematically deviates from attentive behavior.

For model estimation, we suggest Bayesian estimation due to its flexibility in handling complex models (Gelman et al., 2013), with priors assigned to model parameters. These prior values can be informed by theoretical considerations and previous research, or, when prior knowledge is unavailable, can be set as diffuse. In this study, we apply diffuse priors. For the item-specific difficulty and dispersion parameters, 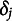 and 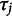 , we use normal priors with 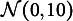 . The latent trait mean for the attentive group is fixed at zero for model identification. The multidimensional latent traits 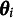 are modeled as 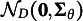 , where the means are fixed to zero for model identification. The covariance matrix 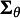 is decomposed into a correlation matrix 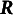 and a vector of standard deviations 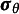 , such that 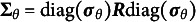 . We place a half-Cauchy prior on each dimension of 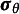 , i.e., 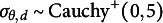 , and a Lewandowski–Kurowicka–Joe (LKJ, Lewandowski et al., 2009) prior on the correlation matrix: 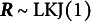 . When 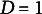 , the prior for latent variable simplifies to 
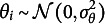
 without correlation structure. For the parameters *m* and *n*, which shape the Beta distribution of careless response patterns, we employ a half-Cauchy distribution 
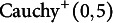
 for accommodating a wide range of values. For the individual attentiveness and carelessness probabilities 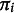 and 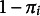 , we employ a Dirichlet prior parameterized as 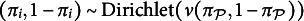 , where 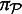 represents the population-level proportion of attentive respondents and 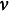 is a concentration parameter (Kemp et al., 2007; Salakhutdinov et al., 2012). For 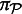 and its counterpart 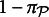 , we employ a diffuse Dirichlet prior 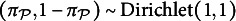 , implying a uniform distribution. For the concentration parameter 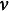 , a half-Cauchy prior with location 0 and scale 5 is used.

## 3 Simulation study 1: Performance of the Beta mixture model

The aim of the simulation study was to evaluate the performance of the proposed model in two aspects: its accuracy in detecting careless respondents exhibiting different behavioral patterns and its estimation accuracy of model parameters of the attentive response model that are adjusted for the occurrence of careless responding.

### 3.1 Data generation and model estimation

We simulated data for a fixed sample size of 300 as it reflects the typical requirement in latent variable analysis (MacCallum et al., 1999). Empirically, this corresponds to the 23rd percentile of sample sizes among the 859 datasets in the Item Response Warehouse (Domingue et al., 2025), representing a realistic lower-bound sample size in applied research. The number of items was set at 10, half of which being negatively worded ( 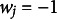 ). This approach enables us to rigorously evaluate the model’s performance under a limited sample size, with the anticipation that as the sample size increases in real-world scenarios, the performance of the mixture model will improve (Zhang et al., 2025). For generating the attentive responses, difficulty parameters and latent factor scores were drawn from a standard normal distribution 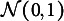 , and dispersion parameters were randomly sampled from a uniform distribution within the [0,3] range following Noel & Dauvier (2007). It is important to note that attentive responses were generated using a unidimensional model, posing a particular challenge for detection. Careless responding is generally easier to identify in multidimensional scales (Ulitzsch, Pohl, et al., 2022), where attentive response patterns tend to exhibit specific structures—such as weaker correlations between items from different scales—that help distinguish them more clearly from careless patterns.

We considered three patterns of careless response behaviors in data generation:

1. Random response pattern at extremes: Responses clustered at the two extremes of the scale, with values randomly selected near either end, following a 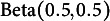 distribution (Figure 4).
2. Overly consistent response pattern: Careless respondents consistently selected values near one end of the slider scale—either the left or the right—regardless of item wording. Participants were randomly assigned to one of these two groups, with responses generated from either 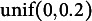 for those favoring the left end or 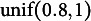 for those favoring the right end.
3. Random response pattern at midpoint: Responses clustered around the midpoint of 0.5, generated from a truncated normal distribution with a mean of 0.5, a standard deviation of 0.25, and bounds set between 0 and 1.

Note that only the first pattern is generated from the Beta distribution. In the latter two data-generating conditions, the Beta distribution can only approximate but not perfectly represent the careless response distribution, presenting a realistic yet potentially challenging scenario. This is especially true for Scenario 2, which (a) simulates a mixture of distinct response preferences, thereby showcasing the model’s capability to “absorb” a mixture of different careless response patterns, and (b) introduces sharp endpoints in both contributing distributions, which are poorly captured by common Beta distributions. While these sharp endpoints may not be entirely realistic—since respondents are likely not able to move their slider uniformly within a precisely defined sub-segment of the scale—we included this scenario to explore the extent to which the careless component of our model can tolerate deviations from a Beta distribution.

Additionally, we varied the proportion of careless respondents ( 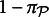 ). We included four low-proportion conditions (0.05, 0.10, 0.15, and 0.25), following prior research on careless response detection, as these levels reflect commonly observed prevalence rates (Kam & Cheung, 2023). To evaluate the model’s performance under more extreme conditions, we also included a higher proportion condition (0.40), where the number of careless respondents approaches that of attentive ones—resulting in five proportion levels in total. For each condition, we generated 100 datasets for replication.^1^

Data generation was conducted using R version 4.3.1 (R Core Team, 2023). Each data set was analyzed with the proposed model using Stan (Carpenter et al., 2017). For model estimation, we ran two Markov chain Monte Carlo (MCMC) chains with 40,000 iterations each, with the first half being employed as burn-in. Model convergence was assessed using the estimated potential scale reduction (EPSR) index (Gelman, 1996), with values below 1.1 indicating satisfactory convergence (Gelman et al., 2013). We evaluated the model performance in terms of estimation and classification accuracy. For model estimation, we saved the posterior means as point estimates. We evaluated the correlation between the true parameter values and the estimated values for parameters generated from random distributions ( 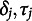 ). We considered the relative bias (i.e., ratio of the difference between the estimated parameter and its true value to the true value) for fixed parameters in each condition ( 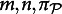 ). Further, the root mean square error (RMSE) was calculated for all parameters to evaluate the accuracy of the parameter estimates.

To assess the classification performance of the proposed model, each respondent was classified based on their individual probability of attentiveness ( 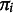 ). We evaluated two methods of classification: 1) a threshold-based classification approach, where 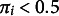 ( 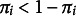 , suggesting the probability of attentiveness is lower than the probability of carelessness) indicates that person *i* is careless and 2) a ranking approach using the population-level proportion of attentive respondents ( 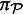 ). Here, we first obtained the 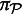 point estimate, then ranked 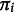 values, and identified the least likely 
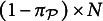
 respondents as careless based on their individual probabilities. This approach allows the classification to reflect the estimate of overall attentiveness. We then evaluated the model’s classification performance using several key metrics: 1) Accuracy, defined as the proportion of respondents correctly classified as either attentive or careless; 2) Sensitivity, defined as the ability of the model to correctly identify attentive respondents, calculated as the proportion of actual attentive respondents correctly classified; 3) Precision, defined as the proportion of respondents classified as attentive who were truly attentive; 4) False positive rate (FPR), defined as the proportion of careless respondents incorrectly classified as attentive; as well as 5) False negative rate (FNR), defined as the proportion of attentive respondents mistakenly classified as careless.

### 3.2 Results

The model exhibited high convergence rates for all conditions ( 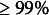 ), except when the careless response pattern was overly consistent. In the overly consistent condition, convergence became increasingly difficult as the proportion of careless respondents rose. Convergence rates exceeded 89% when 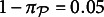 to 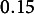 , but declined to 86% and 68% at 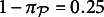 and 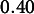 , respectively. This might be due to the fact that the simulated overly consistent careless responses could not be well accommodated by a Beta distribution. To address convergence issues, we increased the number of MCMC iterations for the overly consistent condition with 40% careless respondents. The convergence rate substantially improved—from 68% to 97%—when the number of burn-in iterations was allowed to go up to 100,000. These results suggest that large proportions of careless responses following distributions that challenge the model also pose challenges for model convergence; however, this issue can be mitigated by increasing the number of iterations.

Estimation and classification accuracy were evaluated based on the converged replications. Tables 1 and 2 summarize the classification accuracy based on the threshold 0.5 and the population-level proportion of attentive respondents, respectively. The classification results showed excellent overall performance, with both the threshold-based and population-level proportion methods achieving high accuracy in classifying respondents as attentive or careless. However, when using the threshold method, the FPR reached unacceptably high levels when the proportion of careless respondents ( 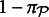 ) was between 0.05 and 0.15. This suggests that when 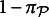 is low, for most careless respondents, the estimated 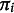 values exceeded 0.5, leading to their misclassification as attentive. Although overall accuracy remained high with consistently strong sensitivity and precision, this is likely due to class imbalance where the majority of respondents are attentive. Moreover, the FPR varied across data-generating scenarios, being low in overly consistent conditions, but higher in random and normal conditions. Under these conditions, the FPR reached as high as 0.477 and 0.456, respectively, when 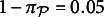 .

**Table 1:** Classification results based on the threshold

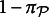	Pattern	Accuracy	Sensitivity	Precision	FPR	FNR
0.05	Beta	0.976	1.000	0.976	0.477	0.000
	Preference	0.994	1.000	0.994	0.119	0.000
	Normal	0.977	1.000	0.977	0.456	0.000
0.10	Beta	0.982	1.000	0.980	0.183	0.000
	Preference	0.993	0.999	0.994	0.060	0.001
	Normal	0.969	0.996	0.971	0.273	0.004
0.15	Beta	0.977	0.999	0.975	0.146	0.001
	Preference	0.992	0.997	0.994	0.035	0.003
	Normal	0.973	0.991	0.979	0.125	0.009
0.25	Beta	0.979	0.996	0.976	0.074	0.004
	Preference	0.980	0.993	0.983	0.060	0.007
	Normal	0.976	0.991	0.969	0.048	0.009
0.4	Beta	0.976	0.991	0.969	0.048	0.009
	Preference	0.956	0.987	0.956	0.089	0.013
	Normal	0.962	0.965	0.971	0.044	0.035

*Note*: Beta = Random responses generated from 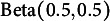 ; Preference = Overly consistent pattern; Normal = Random responses generated from a truncated normal distribution; The term 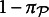 indicates the true population-level proportion of careless respondents; FPR = False positive rate; FNR = False negative rate.

**Table 2:** Classification results based on 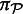

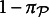	Pattern	Accuracy	Sensitivity	Precision	FPR	FNR
0.05	Beta	0.984	0.985	0.998	0.045	0.015
	Preference	0.969	0.970	0.997	0.053	0.030
	Normal	0.963	0.966	0.995	0.095	0.034
0.10	Beta	0.984	0.985	0.997	0.031	0.015
	Preference	0.961	0.961	0.996	0.037	0.039
	Normal	0.950	0.953	0.990	0.086	0.047
0.15	Beta	0.980	0.985	0.991	0.048	0.015
	Preference	0.955	0.952	0.995	0.031	0.048
	Normal	0.952	0.950	0.993	0.040	0.050
0.25	Beta	0.980	0.986	0.987	0.040	0.014
	Preference	0.943	0.942	0.983	0.054	0.058
	Normal	0.950	0.945	0.987	0.037	0.055
0.4	Beta	0.976	0.986	0.975	0.039	0.014
	Preference	0.916	0.919	0.951	0.089	0.081
	Normal	0.954	0.944	0.979	0.031	0.056

*Note*: Beta = Random responses generated from 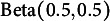 ; Preference = Overly consistent pattern; Normal = Random responses generated from a truncated normal distribution; The term 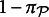 indicates the true population-level proportion of careless respondents; FPR = False positive rate; FNR = False negative rate.

The high FPR observed under the single-threshold (0.5) approach may be attributed to the hierarchical prior structure and the high proportions of attentive respondents. When the two classes are more balanced (e.g., 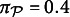 ), the FPR is generally within an acceptable range. In contrast, under imbalanced conditions where 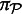 is high, the hierarchical prior structure—with 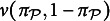 serving as a hyperprior for 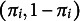 —pulls individual-level estimates 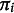 toward higher values (i.e., attentiveness), thereby diminishing the effectiveness of the 0.5 threshold in detecting careless respondents.

The classification method utilizing the population-level proportion ( 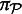 ) exhibited a slightly lower overall accuracy. Nevertheless, it maintained a high level of performance, especially when the proportion of careless respondents was low. Moreover, it provided significantly improved management of the FPR, with FPRs consistently remaining below 0.05 across most conditions, except in several cases where careless responses deviated from the Beta distribution, in which FPRs remained under 0.1. This method also maintained high sensitivity and precision, with sensitivity values ranging from 0.919 to 0.986 and precision consistently above 0.95. Given the ability of the population-level proportion method to better control the FPR while maintaining strong classification performance, we recommend using this approach when the proportion of careless respondents is low.

Table 3 displays results related to the estimation accuracy of the attentive item parameters, the standard deviation of the content trait, and the population-level proportion of attentive respondents. The attentive item parameters (difficulty 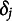 and response variability 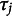 ) were accurately estimated (e.g., high correlations between true and estimated values and small RMSEs). Likewise, the standard deviation of latent trait ( 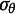 ) was accurately estimated across all conditions, exhibiting very low relative bias and RMSE. To more closely evaluate latent trait estimation, we further examined the factor score estimates for attentive respondents under one selected condition ( 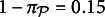 and careless responses were generated from 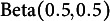 ). We compared the proposed model with the Beta IRM, which does not account for careless responding. Results showed that both models achieved high correlations between estimated and true factor scores, with average correlations across replications of 0.959 for both the proposed model and the Beta IRM. However, the proposed model yielded lower RMSE values (0.288 versus 0.346), indicating it produces more precise estimates with less variability around the true factor scores.

**Table 3:** Estimation accuracy of item parameters and 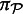

		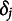		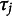		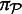		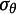	
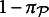	Condition	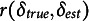	RMSE	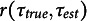	RMSE	RB	RMSE	RB	RMSE
0.05	Beta	0.996	0.088	0.981	0.190	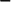 0.011	0.012	0.026	0.053
	Preference	0.996	0.092	0.974	0.224	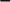 0.026	0.026	0.040	0.063
	Normal	0.997	0.090	0.980	0.200	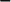 0.027	0.030	0.029	0.053
0.1	Beta	0.996	0.089	0.980	0.203	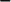 0.009	0.011	0.038	0.065
	Preference	0.995	0.102	0.974	0.230	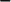 0.034	0.033	0.041	0.070
	Normal	0.996	0.097	0.977	0.216	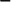 0.035	0.037	0.042	0.067
0.15	Beta	0.996	0.086	0.976	0.217	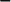 0.004	0.012	0.031	0.059
	Preference	0.996	0.101	0.972	0.245	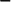 0.042	0.039	0.046	0.067
	Normal	0.996	0.103	0.977	0.216	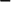 0.041	0.037	0.049	0.067
0.25	Beta	0.995	0.104	0.974	0.232	0.002	0.011	0.034	0.063
	Preference	0.992	0.142	0.930	0.347	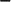 0.038	0.042	0.042	0.086
	Normal	0.995	0.111	0.971	0.236	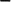 0.040	0.033	0.063	0.081
0.4	Beta	0.994	0.113	0.965	0.263	0.015	0.015	0.043	0.081
	Preference	0.978	0.224	0.869	0.470	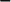 0.019	0.064	0.037	0.116
	Normal	0.994	0.126	0.968	0.258	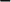 0.033	0.024	0.056	0.082

*Note*: Beta = Random responses generated from 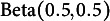 ; Preference = Overly consistent pattern; Normal = Random responses generated from a truncated normal distribution; The term 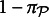 indicates the true population-level proportion of careless respondents; RB = Relative bias; RMSE = Root mean square error.

The accuracy of the estimated population-level proportion of attentive respondents ( 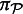 ) depended on the data-generating distribution of careless responses. While the relative bias was essentially zero when careless responses were generated from a Beta distribution, we observed estimates to be downward biased in the other two conditions. That is, under conditions where careless responses cannot be perfectly accommodated by a Beta distribution, researchers might underestimate the proportion of attentive respondents—and, consequently, overestimate the proportion of careless respondents. For instance, when careless responses were generated with an overly consistent pattern, the average estimated proportion of careless respondents was 0.075 and 0.186 in conditions with data-generating proportions of 0.05 and 0.15, respectively. However, we note that the amount of bias was small, such that we deem the model to be sufficiently reliable to roughly gauge the extent of careless respondents in these scenarios. The estimation results of 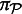 also help explain the variation in classification accuracy across three careless response patterns (Table 2). Classification accuracy based on the population-level cutoff ( 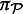 ) was highest when careless responses were generated from a Beta distribution, likely due to the fact that 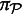 was estimated most accurately under this condition.

**Figure 5:**
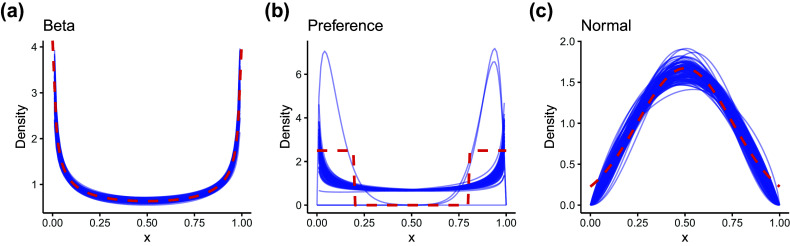
Beta approximation for careless responses. *Note*: Beta = Random responses generated from 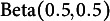 ; Preference = Overly consistent pattern; Normal = Random responses generated from a truncated normal distribution. Blue lines indicate the Beta approximation for careless responses across all simulated datasets, while red dashed lines represent the distribution used for data generation.

Figure 5, comparing data-generating distributions of careless responses and how these were approximated by the proposed model, provides intuition for the sources of variation in estimation accuracy across conditions. The parameter recovery results of these distributional patterns are also presented in Table 4. When careless responses were generated from a Beta distribution (Figure 5a), the distribution of careless responses was well recovered, as was to be expected. The parameter estimates of the Beta distributions closely matched the true values, with low RMSE indicating high estimation accuracy. Note that RMSE values for the shape parameters *m* and *n* could only be computed for the Beta condition, as these parameters are not defined under alternative generating distributions. When careless responses were not generated from a Beta distribution, the proposed model could only approximate but not fully replicate the true distribution of careless responses. For overly consistent responses generated from unif(0, 0.2) or unif(0.8, 1), the estimated distributions typically showed a U-shaped pattern, characterized by underestimated densities around the midpoint and overestimated densities near the scale boundaries (Figure 5b). The parameters of the Beta-approximated distribution showed great variability, as indicated by the large standard deviations in the parameter estimates. Moreover, when data were generated using a truncated normal distribution, the estimated distributions exhibited lighter tails compared to the true distribution (Figure 5c). The Beta approximation in this case resembled a 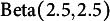 distribution—symmetric around 0.5 and bell-shaped, bearing similarity to a normal distribution.

**Table 4:** Parameter estimation for the careless response distributions

		*m*			*n*		
Condition	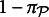	Mean	SD	RMSE	Mean	SD	RMSE
0.05	Beta	0.565	0.110	0.127	0.546	0.082	0.094
	Preference	2.282	4.893	-	1.305	2.867	-
	Normal	2.794	0.842	-	3.036	3.498	-
0.1	Beta	0.507	0.051	0.052	0.509	0.055	0.055
	Preference	1.270	3.046	-	0.968	2.032	-
	Normal	2.584	0.952	-	2.528	0.441	-
0.15	Beta	0.503	0.043	0.043	0.502	0.042	0.041
	Preference	0.957	2.555	-	0.744	1.442	-
	Normal	2.359	0.258	-	2.365	0.269	-
0.25	Beta	0.493	0.029	0.029	0.493	0.028	0.028
	Preference	2.010	4.470	-	0.832	1.510	-
	Normal	2.270	0.170	-	2.28	0.174	-
0.4	Beta	0.492	0.021	0.023	0.496	0.019	0.019
	Preference	1.970	4.260	-	2.000	4.390	-
	Normal	2.160	0.156	-	2.160	0.141	-

*Note*: Beta = Random responses generated from 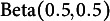 ; Preference = Overly consistent pattern; Normal = Random responses generated from a truncated normal distribution; The term 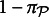 indicates the true population-level proportion of careless respondents; Mean = Averaged estimates across all converged replications; SD = Standard deviation of these estimates; RMSE is reported only for the Beta condition, where the true values of the Beta distribution are known.

## 4 Simulation study 2: Comparison with the mixture CFA model

As mentioned in the introduction, the unique characteristics of VAS data render previous mixture modeling approaches for careless respondent detection unsuitable, as their underlying assumptions (e.g., normality) may not hold for VAS data. To further demonstrate this, we conducted a simulation study to evaluate the limitations of a mixture CFA model for VAS data. Using the simulated datasets from Simulation Study 1, we applied a mixture CFA model (Roman et al., 2024; Zhang et al., 2025) to the data: 
(5)

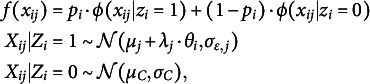

where 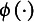 represents the normal density function. The item parameters 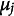 , 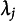 , and 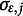 represent intercepts, item loading, and residual standard deviations in the attentive group. It is important to note that, because the simulation design is based on a unidimensional model, 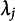 is a scalar rather than a vector. In the mixture CFA model, 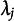 is estimated using a half-normal prior with a mean of 0 and a standard deviation of 10. Its direction (positive or negative) is constrained based on the item’s wording. Careless responses are assumed to stem from a normal distribution 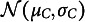 with a common mean and standard deviation. The remaining simulation settings, such as the number of iterations, were kept consistent to ensure comparability.

The model convergence rate significantly declined under the overly consistent careless response condition compared to the proposed model (51% versus 93% when 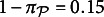 ). This decline is likely due to careless responses strongly deviating from normality, which might impair the mixture CFA model’s ability to differentiate the two groups. In contrast, for the other two conditions involving random careless responses, the model showed better convergence rates (over 80%).

Based on the converged replications, Table 5 summarizes the classification results of the mixture CFA model when 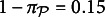 . Notably, as 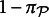 decreases, the classification performance worsens, with the FPR reaching 0.7 under the threshold method. For brevity, we focus on the conditions where 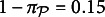 . Table 5 highlights that the mixture CFA model fails to classify attentive and careless respondents effectively in terms of accuracy, FPR, and FNR. It is worth noting that when careless responding was generated using a truncated normal distribution, the mixture CFA model performed better than under other conditions. However, it still fell short of achieving acceptable levels of classification error rates. Although the generated and estimated distributions of careless responses were closely aligned in this scenario, the model’s limitations likely stem from a violation of its assumptions for the attentive group.

**Table 5:** Classification results of the mixture CFA model when 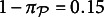 .

	Pattern	Accuracy	Sensitivity	Precision	FPR	FNR
Threshold	Beta	0.817	0.829	0.956	0.249	0.171
	Preference	0.759	0.834	0.880	0.669	0.166
	Normal	0.905	0.906	0.982	0.105	0.094
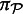	Beta	0.767	0.736	0.985	0.058	0.264
	Preference	0.658	0.648	0.923	0.287	0.352
	Normal	0.835	0.811	0.994	0.033	0.189

*Note*: Beta = Random responses generated from 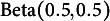 ; Preference = Overly consistent pattern; Normal = Random responses generated from a truncated normal distribution; The term 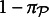 indicates the true population-level proportion of careless respondents; FPR = False positive rate; FNR = False negative rate.

## 5 Empirical illustration

We applied the model to empirical data with three objectives: (a) to illustrate the practical application of the proposed model for pinpointing careless respondents, (b) to assess the impact of adjusting for careless respondents on parameter estimation, and (c) to compare the prevalence of careless behaviors between VAS and Likert data. The data used in this analysis come from a study that collected responses through two distinct scale formats—VAS and Likert—across three questionnaires, labeled A, B, and C, that differed in which scales and formats were administered (Kalistová & Cígler, 2021). These questionnaires measured two constructs: height, which captures individuals’ perceptions and experiences related to their physical stature (Tancoš, 2018), and autonomy, which reflects the extent to which individuals feel free to make their own decisions and express their preferences (Johnston & Finney, 2010). We utilized data from questionnaire C to demonstrate the application of the proposed model, as it includes responses for both the height and autonomy scales in both VAS and Likert formats, allowing for a direct comparison between these response formats.

Questionnaire C is organized into four sequential blocks: 1) Height assessed using a VAS, 2) Autonomy assessed using a VAS, 3) Height assessed using a Likert scale, and 4) Autonomy assessed using a Likert scale. The Likert scale used in this study is a 5-point scale. The height scale contains 11 items (labeled h1–h11), of which 6 are negatively-worded, while the autonomy scale includes seven items (labeled a1–a7) with 3 negatively-worded items. Rows containing complete missing data in either the VAS or Likert scale were excluded from the analysis, resulting in a sample size of 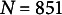 .

We applied four models to analyze the data. For the VAS data, we used the Beta IRM (Noel & Dauvier, 2007), which assumes all responses are attentive and incorporates two factors—height and autonomy. This was compared to the proposed model, which is designed to identify careless respondents. For the Likert scale data, we applied the generalized partial credit model (gPCM), which does not account for careless responding. We also included an ordinal mixture model (Ulitzsch, Pohl, et al., 2024), which was designed to detect careless respondents in ordinal data. The ordinal mixture model follows a similar approach as the proposed model to disentangle attentive from careless patterns; however, it is designed for ordinal data and utilizes the gPCM instead of the Beta IRM for the attentive class. For the careless class, the model estimates marginal category probabilities of inattentively choosing a given category over all types of careless behavior. Specifically, the probability of observing response category 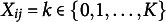 is given by: 
(6)

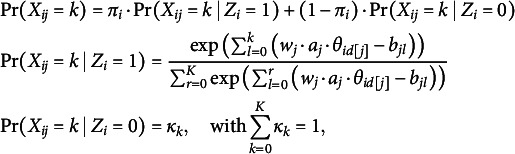

where 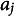 is the discrimination parameter, controlling how strongly the item differentiates between respondents with different trait levels and 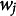 is a pre-specified wording parameter. The step difficulty parameter 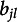 governs the relative difficulty of selecting higher response categories. Note that 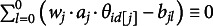 . For more detailed information regarding the ordinal mixture model, please refer to Ulitzsch, Pohl, et al. (2024).

Prior settings for the proposed model and Beta IRM were the same as in the simulation study. However, unlike the simulation study, the empirical study modeled two factors: height and autonomy. To estimate the correlation between these factors, an LKJ (Lewandowski et al., 2009) prior with a shape parameter of 1 was used for the factor correlation matrix. For the ordinal mixture model and gPCM, we assigned weakly informative priors to the discrimination ( 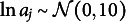 ) and threshold parameters ( 
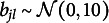
 ). The inattentive response probabilities 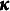 are drawn from a Dirichlet prior with all parameters equal to 1. The priors for estimating the class probabilities in the ordinal mixture model were consistent with those specified for the proposed model in the simulation study. The variances of the latent traits were fixed to 1 for model identification in both the ordinal mixture model and gPCM. In all models used in the empirical study, two MCMC chains were generated with 10,000 iterations, with the first half designated as burn-in iterations. All models reached convergence, as indicated by EPSR values of less than 1.1 (Gelman, 1996).

### 5.1 Investigating careless responding in VAS data

**Figure 6:**
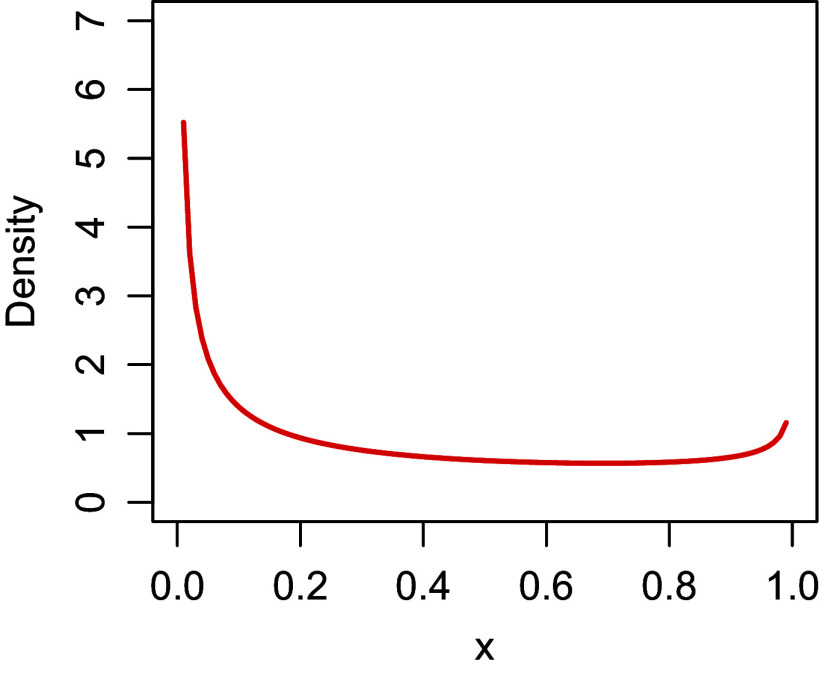
Beta(0.388, 0.728): Model-implied distribution of careless responses in the empirical illustration.

The population-level proportion of careless respondents ( 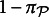 ) for the VAS was 0.08, with a 95% credibility interval of [0.069, 0.092]. The parameters for the Beta distribution of careless responses, with 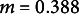 (95% CI: [0.357, 0.420]) and 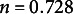 (95% CI: [0.640, 0.823]), indicated that, marginally, careless respondents tended to select the left side of the slider scale (Figure 6).

Given results from the simulation study (Figures 1 and 2), we identified the careless respondents using 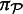 . We ranked the 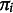 values and identified the least likely respondents as careless by selecting the bottom 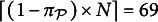 individuals according to their individual probabilities.

**Figure 7:**
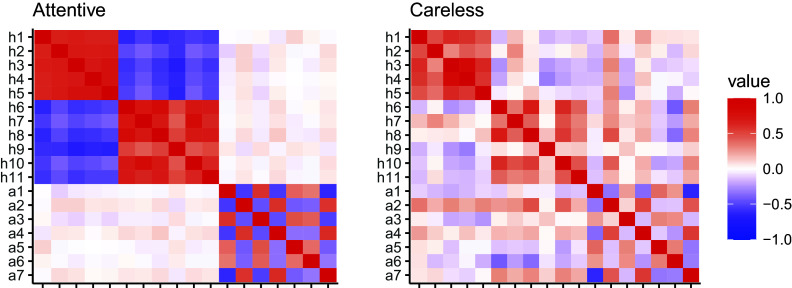
Inter-item correlations for attentive and careless groups for VAS. *Note*: The item labels “h” and “a” represent the respective scales for “height” and “autonomy.”

Figure 7 compares the item correlations within and across the two scales for the attentive and careless groups. Responses from the attentive group showed strong correlations within both the height and autonomy scales. The negatively-worded items exhibited negative correlations with the positively worded items within the same scale. This demonstrates that attentive respondents answered according to item wording, aligning with the scale design. The correlations between items across the two scales were very low, highlighting the distinctiveness of the height and autonomy measures. Additionally, the low factor correlation that was not credibly different from zero further suggested the distinct separation between the height and autonomy constructs ( 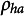 in Table 6).

**Table 6:** Summary of estimates for VAS data

	Beta IRM			The proposed model		
Parameter	Est	2.5%	97.5%	Est	2.5%	97.5%
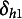	0.723	0.615	0.826	0.771	0.638	0.896
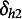	1.620	1.509	1.733	1.699	1.561	1.832
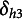	1.268	1.160	1.372	1.308	1.183	1.431
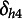	1.254	1.146	1.358	1.259	1.142	1.377
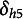	1.436	1.331	1.548	1.525	1.399	1.653
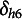	0.865	0.755	0.969	0.874	0.752	1.000
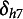	1.460	1.351	1.569	1.486	1.362	1.618
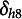	0.509	0.407	0.619	0.487	0.368	0.611
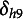	-0.599	-0.718	-0.483	-0.737	-0.861	-0.607
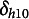	1.216	1.102	1.325	1.190	1.060	1.321
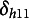	0.494	0.393	0.596	0.366	0.245	0.492
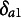	-1.404	-1.484	-1.322	-1.467	-1.547	-1.384
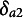	0.630	0.552	0.710	0.752	0.669	0.836
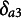	-1.396	-1.477	-1.314	-1.487	-1.577	-1.396
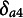	0.597	0.523	0.673	0.656	0.569	0.739
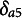	-0.725	-0.803	-0.646	-0.845	-0.929	-0.763
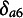	-0.610	-0.694	-0.529	-0.774	-0.862	-0.685
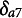	1.094	1.021	1.166	1.191	1.106	1.276
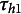	0.763	0.595	0.940	1.570	1.355	1.779
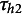	0.841	0.670	1.018	1.678	1.451	1.898
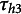	1.336	1.146	1.509	2.314	2.088	2.546
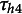	1.212	1.034	1.390	2.058	1.831	2.291
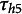	1.025	0.848	1.198	1.918	1.686	2.137
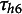	1.159	0.971	1.336	1.927	1.705	2.137
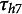	0.941	0.757	1.119	1.707	1.505	1.916
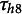	1.092	0.911	1.277	1.986	1.764	2.199
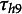	0.591	0.412	0.759	1.455	1.229	1.669
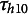	0.652	0.472	0.823	1.341	1.108	1.548
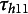	0.978	0.805	1.169	2.157	1.904	2.388
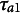	2.154	1.961	2.329	2.737	2.472	2.984
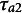	1.493	1.293	1.674	2.489	2.229	2.744
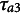	2.104	1.903	2.297	2.780	2.559	3.015
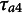	2.067	1.867	2.272	2.978	2.711	3.235
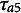	1.755	1.568	1.950	2.626	2.360	2.884
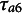	1.217	1.023	1.386	2.038	1.786	2.288
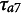	2.173	1.982	2.363	3.216	2.965	3.484
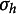	1.197	1.137	1.261	1.471	1.397	1.551
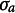	0.759	0.718	0.805	0.897	0.848	0.952
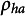	0.032	-0.039	0.106	0.054	-0.029	0.134
*m*				0.388	0.357	0.420
*n*				0.728	0.640	0.823
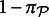				0.080	0.069	0.092

*Note*: The 2.50% and 97.50% columns indicate the lower and upper bounds of the 95% credible interval. The symbols 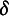 and 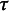 represent item difficulty and dispersion, 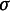 and 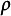 denote the standard deviations of the height and autonomy factors and the correlation between them, *m* and *n* indicate the Beta distribution shape for careless responses, and 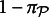 is the estimated population-level proportion of careless respondents.

In contrast, the careless group displayed a more chaotic correlation structure. There were weaker and more inconsistent correlations within the same scale, and the negatively-worded items did not display the expected negative correlations. This suggests that careless respondents tended to ignore item wording and might not have distinguished between scales, leading to a breakdown in the expected correlation structure.

### 5.2 Impact of disregarding careless respondents on parameter estimates

**Figure 8:**
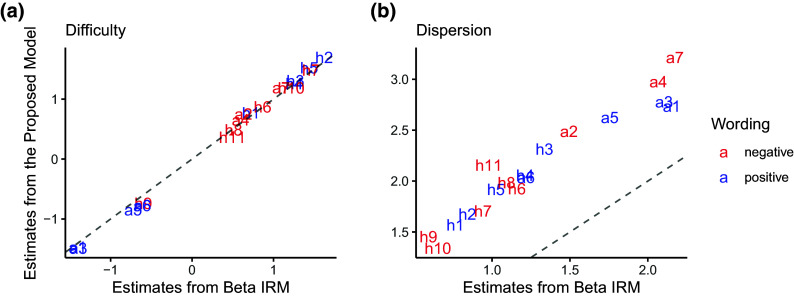
Item parameter estimates for VAS obtained from different models. *Note*: The dashed line shows where the obtained estimates are equal; the labels “h” and “a” represent the item parameters of the “height” and “autonomy” scales.

**Figure 9:**
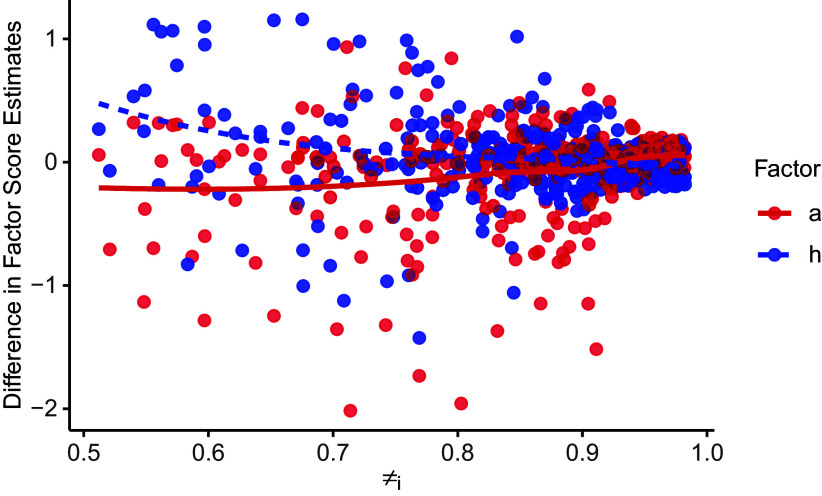
Difference in factor score estimates. *Note*: 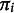 denotes the probability that person *i* belongs to the attentive group; the labels “h” and “a” represent the respective factors of “height” and “autonomy.”

Results of parameter estimates and credible intervals for the difficulty and dispersion parameters are provided in Table 6. After accounting for the careless respondents, the factor correlation ( 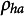 ) slightly increased from 0.032 (95% CI: [ 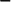 0.039, 0.106]) to 0.054 (95% CI: [ 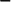 0.029, .134]), though both estimates remained not credibly different from zero. Figure 8 displays a comparison of the estimates between the proposed model and the Beta IRM that does not take careless responding into account. Generally, both models yielded similar estimates for item difficulty parameters ( 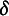 ) across the height and autonomy scales. However, the proposed model consistently yielded higher estimates for item dispersion parameters. As depicted in Figure 2, increased dispersion implies enhanced item informativeness, indicating an improvement in item psychometric properties upon accounting for careless responses. Figure 9 further compares the factor score estimates between the proposed model and the Beta IRM, plotted against person-level posterior attentiveness probability obtained from the proposed model. As can be seen, differences in factor score estimates were especially pronounced for individuals with lower attentiveness probabilities, while the two models yielded similar factor score estimates when the posterior attentiveness probability was high.

### 5.3 Comparison between VAS and Likert scale

With a population-level estimate of 0.047 (95% CI [0.035, 0.061]), the proportion of careless respondents in the Likert scale data was lower than that observed in the VAS data. To evaluate whether the difference was indeed credibly different from zero, we estimated it as a derived parameter (0.034; [0.015, 0.052]). Partially mirroring results obtained for the VAS, marginally, careless respondents tended to prefer the lower two ( 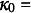 0.268, [0.160, 0.422]; 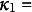 0.260, [0.136, 0.389]) as well as the middle upper categories ( 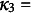 0.239 [0.178, 0.304]).

There was some overlap in the identification of careless respondents between the VAS and the Likert scale. Table 7 presents the contingency table for classification in the VAS and Likert scale data. While some respondents were identified as careless on only one of the scales, an overall agreement of 90.71% between the two scales reflects a high level of consistency in the classifications. Cohen’s 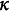 (0.23, 95% CI: [0.12, 0.34]) indicated a fair level of agreement between the Likert and VAS classifications (Cohen, 1960). A chi-square test suggested a significant association between the respondent classifications of attentive and careless across two scales ( 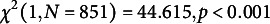 ).

**Figure 10:**
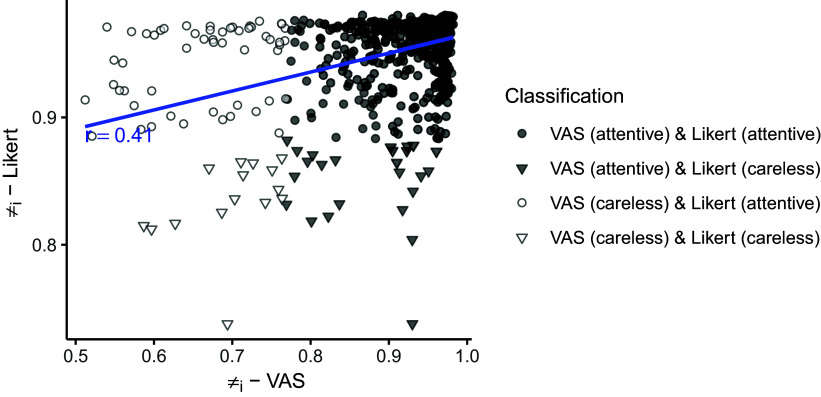
Comparison of individual probabilities ( 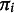 ) between VAS and Likert scale.

Figure 10 compares the individual probabilities of belonging to the attentive group across the two scales. The results showed a moderate correlation between the individual probabilities ( 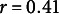 ). Notably, if a classification threshold of 0.5 was applied, none of the respondents would be identified as careless for both scale formats. This finding aligns with the simulation results and suggests that the 0.5 threshold might have difficulty identifying careless respondents when the population-level proportion of such respondents is low.

**Table 7:** Contingency table for classification.

		VAS	
		Attentive	Careless
**Likert**	**Attentive**	757	54
	**Careless**	25	15

**Figure 11:**
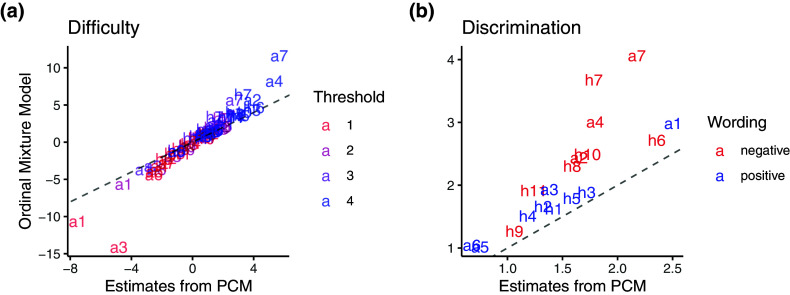
Item parameter estimates for Likert scale obtained from different models. *Note*: The dashed line shows where the obtained estimates are equal; the labels “h” and “a” represent the item parameters of the “height” and “autonomy” scales.

Figure 11 shows the estimated threshold and discrimination parameters between the ordinal mixture model and gPCM. Consistent with the comparison between the proposed model and the Beta IRM (Figure 8), most of the threshold estimates remained very similar before and after accounting for careless responders. The discrimination parameters, however, increased after considering careless responses in the mixture model. Notably, negatively worded items exhibited a greater increase in discrimination estimates. Moreover, the factor correlation in the Likert scales also slightly increased from 0.039 (95% CI: [ 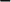 0.037, 0.113]) to 0.055 (95% CI: [ 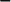 0.017, 0.139]); however, both estimates remained not credibly different from zero.

## 6 Discussion

We proposed a Beta Mixture IRM designed to identify careless respondents in VAS data. VASs are frequently used in fields like psychology, education, and health research to capture subjective experiences with greater sensitivity than ordinal scales. However, careless responses can undermine this sensitivity, skewing results, and compromising the validity of findings. The proposed model allows to detect and account for these responses.

In our simulation study, the proposed model effectively distinguished between attentive and careless respondents. It performed best in both classification and parameter estimation when careless responses followed the assumed Beta distribution. When the distribution of careless responses deviated from the Beta distribution used to “absorb” careless responses, although the proportion of careless respondents was slightly overestimated, the amount of bias was, in our view, small enough to still render the proposed model a useful tool to gauge the overall extent of careless responding in the data. Simulation results still demonstrated high classification accuracy across these varied scenarios. These results suggest that the proposed model provides a flexible and practical framework for capturing a broad range of careless response behaviors. Moreover, item parameters were well recovered across all conditions, indicating that the proposed model is an effective tool to adjust for careless responding when estimating substantive parameters of interest. Furthermore, the simulation results underscored the limitations of existing mixture CFA models, which are challenged by violations of normality in both attentive and careless responding.

Our empirical results further demonstrated the practical applicability of the proposed model by highlighting distinct correlation patterns between attentive and careless respondents. Attentive respondents showed strong, consistent correlations within the same scale, with negatively-worded items correlating negatively as expected, whereas careless respondents displayed weak and inconsistent correlations, indicating a disregard for item wording. When compared with the Beta IRM that assumes all responses to be attentive, accounting for careless respondents yielded higher dispersion estimates for VAS items, which suggests that once careless respondents are identified and accounted for, the item information improves.

The unique design of the empirical data, administering the same scales with both VAS and Likert scale formats, further allowed us to conduct initial evaluations on differential scale-specific prevalences of careless responding. In the data at hand, the Likert scale indicated a lower proportion of careless respondents. However, while the probability of attentiveness was moderately correlated between the VAS and Likert scales, some respondents were classified as careless in only one format. A possible explanation for this finding may be that some respondents may engage more effectively with certain response formats. For instance, Dourado et al. (2021) found that most respondents preferred the Likert scale in assessing facial pleasantness, as they found it easier to express their opinions compared to the VAS. Future research could investigate whether allowing respondents to choose their preferred response format (Kutscher & Eid, 2024) might help reduce careless responding. It should be noted, however, that the within-subject design used in this study does not allow to disentangle order effects from scale effects, leaving room for alternative interpretations. For instance, respondents might have been influenced by the order in which the response formats were presented, rather than engaging differently with the scales themselves. Additionally, it is possible that the models did not adequately uncover careless responding, which could account for some of the observed discrepancies. Future research is encouraged to address these design limitations, further evaluate the robustness of the proposed model, and explore attentiveness differences across different scale formats.

### 6.1 Limitations and future directions

Practically, the proposed model offers a safeguard for researchers using VASs to ensure their data accurately reflects the constructs being measured. There are several promising directions for future research to further enhance careless respondent detection and survey data reliability. One such direction is the integration of collateral information, such as response times, to enhance the detection of inattentive behaviors. Response time has been shown to correlate with engagement, with shorter times often reflecting careless responding. Adding this diagnostic layer could create a more robust approach to identifying carelessness, ultimately improving the reliability and validity of survey data (Ulitzsch, Pohl, et al., 2022; Zhang et al., 2025). We suspect that incorporating response time information into the proposed model may be especially advantageous when the distribution of careless responses deviates from a perfect fit with the Beta distribution.

Note that the proposed model identifies careless responding at the respondent level. However, inattentiveness may vary across specific items or sections of a questionnaire. For instance, respondents may start attentively but be more prone to careless responding later in the questionnaire. This raises the need for future research to identify careless responding at the level of single item responses, enabling researchers to pinpoint areas within a survey that may be more prone to inattention. A straightforward way to do so would be to integrate the proposed models with previous model developments that allow for varying attentiveness across the questionnaire but, so far, have only been available for ordinal and continuous, unbounded item responses (Roman et al., 2024; Ulitzsch, Pohl, et al., 2022; Ulitzsch, Yildirim-Erbasli, et al., 2022).

Furthermore, the model’s reliance on assumptions about attentive behavior poses a limitation; if the attentive response model is mis-specified (e.g., a uni- instead is assumed but a multidimensional model holds), the accuracy of detecting careless responses could be compromised (Vogelsmeier et al., 2024). Future research is recommended to evaluate the performance of the proposed model under these conditions. Additionally, the data generation setting considered in the simulation study ( 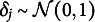 ), which results in highly heterogeneous items, is particularly favorable for the model. It is important to note that the model’s performance may decline when item heterogeneity is reduced.

Another important consideration is that we presented the model with a deliberately misspecified careless component model, designed to “absorb” different careless response patterns present in the data, described marginally by a common Beta distribution. If researchers want to disentangle different careless response patterns, they could specify the model with an empirically determined number of careless classes, each with its own Beta distribution. Note, however, that this approach may be challenging and unstable to estimate when some specific careless classes are very small, i.e., when a specific careless response pattern is only exhibited by very few respondents. Future research may compare this approach with the residual class approach taken in the present study and derive guidelines on how to choose between these different approaches.

We have introduced and evaluated the proposed model for cross-sectional data. Nevertheless, we see its highest applicability in the context of longitudinal ecological momentary assessments, where VASs are a common scale of choice to capture real-time changes in participant experiences (Haslbeck et al., 2024). As participants are at risk of becoming inattentive over time due to the repetitive nature and increased respondent burden of ecological momentary assessments (Eisele et al., 2022; Ulitzsch, Nestler, et al., 2024; Ulitzsch, Viechtbauer, et al., 2025; Vogelsmeier et al., 2024), future research could integrate the mixture model with dynamic models to track attentiveness over time, which, so far, have only been developed for Likert-type scales (Vogelsmeier et al., 2024). This extension would be particularly valuable for health and psychological well-being studies, where real-time, accurate data is critical for understanding changes in subjective experiences.

## Data Availability

A preprint of this manuscript is available at https://doi.org/10.31219/osf.io/tp6df_v1 and the code for the empirical study can be accessed through this OSF Project.

## Footnotes

1 We conducted a test run with 200 replications for one simulation condition. The results were highly consistent with those from the 100-replication run. Given this consistency and the high computational cost (around 1-hour per dataset on an M2 Max chip), we retained 100 replications for efficiency.
